# Genetic Counseling in Direct-to-Consumer Exome Sequencing: A Case Report

**DOI:** 10.1007/s10897-014-9737-0

**Published:** 2014-10-01

**Authors:** Saskia van den Berg, Yaoqing Shen, Steven J. M. Jones, William T. Gibson

**Affiliations:** 1 Department of Medical Genetics, University of British Columbia, Vancouver, BC Canada; 2 Child and Family Research Institute, A4-151, Bay 17, 950 West 28th Avenue, Vancouver, BC V5Z 4H4 Canada; 3 Canada’s Michael Smith Genome Sciences Centre, British Columbia Cancer Agency, Vancouver, BC Canada

**Keywords:** Genetic Counseling, Rare Variant, Polycystic Ovarian Syndrome, Exome Sequencing, Familial Hypercholesterolemia

## Introduction

As the health care system moves further toward enhanced patient empowerment, many members of the general population are seeking medical answers for themselves in their genome. Direct-to-consumer (DTC) companies offer genetic testing that promises to establish ancestry and predisposition to traits, diseases and conditions (Harris et al. 2013). DTC companies predominately offer panel-based testing, which interrogates single nucleotide polymorphisms (SNPs) in or near specific genes. Some panels target ancestry; others target SNPs that have been associated statistically with disease. The true predictive value of panels that incorporate numerous SNPS into mathematical risk modeling is unknown, particularly when considering the small proportion of the overall heritability of a trait that is accounted for by these genetic variants (McCarthy et al. 2008). Perhaps in response to this and to concerns articulated by regulatory authorities, some companies have left the DTC medical testing market (Kalf et al. 2014). Other companies are looking to expand their services to more comprehensive genetic testing, such as whole exome sequencing (WES).

Although high-throughput genetic testing such as whole exome sequencing and whole genome sequencing (WGS) are typically offered only upon the request of a physician who is caring for the patient (Yang et al. 2013), we were recently consulted by a woman who had had WES performed by 23andMe on a private-pay, direct-to-consumer basis as part of the company’s own in-house research protocol (the Exome 80X Pilot Program). Results of physician-ordered WES and WGS are returned to the requesting physician with an interpretation from the lab, though the physician must use his or her own professional judgment in making the final decision on whether to act on any variants flagged as “actionable” by the testing laboratory (Sturm and Manickam 2012). This woman first consulted us at a time when she had access to her raw data, but had not yet received the research report from 23andMe. Subsequent to our analysis, she did receive a report that flagged some (but not all) of the variants that we had identified, and listed several rare variants in other genes of medical interest. Thus, we had the opportunity to interpret what we considered medically actionable in her exome and compare our own results to those of the company, which the consultand provided to us after we had completed our own research-based analysis and Sanger validation studies.

To our knowledge, WES is still new to the DTC market and not yet widely available. Although the coding exome represents only 1 % of the genome, it is the best-understood part of the genome and is believed to contain 85 % of disease-causing variants (Choi et al. 2009). WES has the potential to be more informative than panels, specifically because of the inclusion of rare DNA variants that would have a higher allele-specific effect size (and thus higher predictive value for disease). However, the information generated by WES is difficult and time-consuming to interpret due to its sheer volume and complexity (Dewey et al. 2014). Furthermore, Variants of Uncertain Significance (VUS - genetic variants not previously identified or on which there is little population or functional data) are found in every clinical and research exome. Thus, any detailed WES report is likely to contain several rare variants that may be disease-causing in the heterozygous state (dominant pathogenic variants), may be disease-causing in the homozygous or compound heterozygous state (recessive pathogenic variants), or may be disease-causing in the hemizygous state (X-linked recessive pathogenic variants) as well as those that are rare but may not be causative of disease at all (truly benign variants). There are also likely to be many rare variants that modify disease risk to a small extent, but due to the low power of any study to quantify the effect of a rare, mildly deleterious allele, these will not be discussed explicitly here.

An important ethical challenge when sequencing the exome is the possibility of “incidental findings” – sometimes defined as actionable variants discovered as a result of genetic testing that are unrelated to the original reason for the test (Lohn et al. 2013). The ACMG recently published (Green et al. 2013) and clarified (ACMG 2013) recommendations for reporting such findings, suggesting that clinical laboratories have the ethical responsibility to look for and report variants in 57 genes that have been associated confidently with disease. Though these best-practice guidelines would apply to certified medical testing labs, they do not apply explicitly to research testing, or to DTC WES testing that is not licensed as a clinical test. Nonetheless, these guidelines do provide the closest approximation of a consensus within the field regarding best practices in reporting, when confronted by one or more incidental findings.

The “early adopters” of DTC genetic testing have been reported to be relatively well-educated and to have high confidence in their ability to navigate the health care system and to understand genetics (Gollust et al. 2012; McBride et al. 2009). They tend to be concerned about their risk of a particular disease, or motivated more by curiosity. Early adopters often turn to healthcare professionals, such as physicians, to help them interpret the results and/or for clinical action (such as further follow up or health interventions) in response to the genetic testing report (Gollust et al. 2012).

When faced with a patient requesting interpretation and additional clinical tests based on a DTC company’s genetic testing report, many healthcare professionals are uncertain regarding the specifics of their fiduciary duty. Primary care practitioners may not possess the knowledge to interpret the results fully, nor to make a clinical decision based on the reports. In a study of genetic counselors and clinical geneticists in Australia, it was found that only 7 % were confident in their ability to interpret and explain DTC genetic testing results (Brett et al. 2012). While the study included SNP panels, whole genome and exome sequencing as well as single-gene sequencing, it is clear healthcare professionals are unprepared for patients wishing to discuss their results. Furthermore, because the predictive power of these test results is often not as strong as the DTC reports suggest (Wade and Wilfond 2006), the effectiveness (i.e. clinical utility) of interventions based upon DTC genetic results has not yet been established. This uncertainty places genetic counselors and other healthcare professionals in a difficult position. Should they interpret the results, and if so, how do they know when they have extracted all relevant information from the report? How far into the primary literature must they go to interpret a panel-based test, and must they provide a specific interpretation for each rare variant, if such a list is also provided?

The following case study describes the journey of an individual woman who sought exome sequencing on a DTC basis, and then sought subsequent data analysis and interpretation. We discuss the challenges of interpreting exome data in the context of a complicated medical history of common, complex disease, and of providing genetic counseling on rare variants to such individuals. The patient provided written informed consent for her personal data to be interpreted on a research basis, and for her case to be presented in this journal.

## Case History

### Medical History

The individual is of Sinhalese descent and is currently 53 years of age, with a Bachelor’s degree from a major Canadian university. She recalls that her medical problems began when she was approximately 26 years old. She was prescribed flutamide in her early 30s for hirsutism. While on it, she experienced unusual personality changes and a drastic loss of hand-eye coordination. After 6 months of treatment, she stopped the drug and the personality changes resolved. However, she does not believe her hand-eye coordination recovered fully. Subsequently, she has experienced additional adverse drug reactions to multiple medications, including adverse reactions to drugs she had previously tolerated well such as statins.

She did not recall the exact age at which she was diagnosed with polycystic ovarian syndrome, but had a right ovarian cystectomy her early 40s, and 2–3 years later, a full hysterectomy and right oophorectomy for menorrhagia. She had also been followed for anxiety, thought to be related to a difficult childhood. She stated that she had been found to have elevated testosterone levels and decreased estrogen and progesterone levels since her oophorectomy, though primary documentation was not available. She had been diagnosed with hyperlipidemia in her early 30s, and mild type I von Willebrand’s disease in her late 40s, and was documented to have low levels of von Willebrand antigen of 0.36 IU (reference range >0.50 IU), ristocetin cofactor activity of 0.30 IU (ref range >0.50 IU), and a factor VIII level of 0.40 IU (Ref range 0.5-1.5 IU). Around the same time, she was diagnosed with moderate-to-severe obstructive sleep apnea-hypopnea. Hepatic ultrasound revealed an enlarged liver with an echotexture consistent with fatty infiltration, though she did not report a history of high alcohol intake and denied tobacco smoking or the use of illicit drugs. In August of the year prior to her consultation with us, her total cholesterol was 7.48 mmol/L (288.8 mg/dL), with LDL 5.4 mmol/L (208.5 mg/dL), HDL 0.94 mmol/L (36.3 mg/dL) and triglycerides 2.5 mmol/L (221.2 mg/dL) and her hemoglobin A1c was elevated at 6.6 %. Healthy levels of lipids are total cholesterol <5.2 mmol/L (i.e. <200.8 mg/dL), LDL <3.4 mmol/L (i.e. <131.3 mg/dL), HDL >1.3 mmol/L (i.e. >50.2 mg/dL) and triglycerides <1.7 mmol/L (i.e. 88.5 mg/dL) (Genest et al. 2009). Repeat blood work done prior to her clinic visit showed ApoB at 1.57 g/L (healthy range <1.25) and hemoglobin A1c at 6.9 %. Taken together, her ApoB and lipid levels were consistent with hyperlipidemia and her hemoglobin A1c was in the range diagnostic for type 2 diabetes (Miremadi et al. 2002; Wilson et al. 1998). At the time of these tests, she was not taking any lipid or glucose-lowering drugs because she had not found any that she was able to tolerate, but she was taking ramipril 10 mg daily to lower her blood pressure. She had tried many natural remedies in an effort to alleviate her discomfort and had also experienced adverse reactions to some of these remedies. She lives alone and does not exercise regularly. Her medical problems had been deemed significant enough to merit a disability pension.

### Testing by Direct-to-Consumer Companies

Concerned in particular about her unusual series of adverse drug reactions, and a history of health problems similar to her own among several family members (Table I), she located a private company that offered a “detoxigenomic panel” of SNP polymorphism testing for drug metabolizing enzymes expressed in the liver. The test reported that she might be missing some of these enzymes, though the report was very unclear; only generic recommendations were provided and the section on “Physician Recommendations” for each SNP series was left blank. The patient’s interest in genetic testing grew and she became aware of 23andMe through a television series on genealogy that featured a segment on genetics. By this time she was, in her own words, “desperate for answers” for her family. She initially had the company’s Personal Genome Service SNP panel done, and later paid $999 US privately for personal exome sequencing when 23andMe offered this to its clientele as part of a research pilot project in late 2011 (“Exome 80x Pilot project” 27 Sept 2011). Four months after she requested the test, the company provided her with the raw data files (in .bam format).

**Fig. 1:**
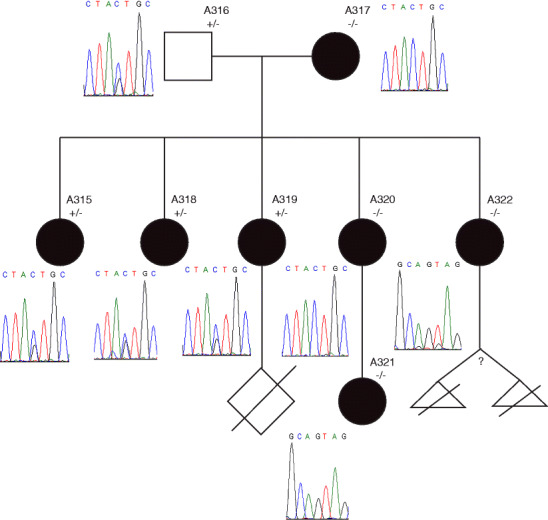
CYP19A1 mutation validation. The mutation was not found to co-segregate with the phenotype of Polycystic Ovarian Syndrome (*black circles*). Unaffected individuals are represented by *unfilled circles*. The proband is indicated by the *black arrow*

**Table I:** Clinical symptoms of family members shown in Figure 1

	A315	A316	A317	A318	A319	A320	A322
Relation to the patient	self	father	mother	sister	sister	sister	sister
High blood pressure	X	X	X	X	X	X	X
High cholesterol	X	X	X	X	X	X	X
“Hormone problems”	X		X	X	X	X	X
Migraines		X		X	X	X	
Polycystic ovarian syndrome (PCOS)	X		X	X	X	X	X
Chronic pain	X	X		X	X	X	X
Anxiety/Depression	X	X	X	X	X	X	X
Hirsutism	X			X	X	X	X
Insomnia	X	X		X	X	X	
Other	-Enlarged fatty liver -Adverse reaction to numerous medications -Type 2 diabetes -Von Willebrand Disease	-Type 2 Diabetes -Adverse reaction to numerous medications -History of myocardial infarction and quadruple bypass surgery -History of Polycythemia without specific diagnosis -Prostatic hypertrophy -“Kidney Weakness”	-History of myocardial infarction and Coronary artery narrowing -Memory difficulties	-Fibromyalgia -“Kidney problems” -Osteoarthritis	-Gastrointestinal Ulcers -“Liver problems,” -Tinnitus	-Anorexia-Bulimia	- Irritated bowel syndrome (IBS) -Cholecystectomy for gallstones -Kidney stones -Bilateral Cornea transplant

The patient was aware that the company would not be providing genetic counseling, but admitted that she did not truly understand the results and their implications. Lacking the appropriate software to open the .bam files, she contacted various bioinformaticians through the internet, including her local Genome Sciences Centre (Y.S. and S.J.), who agreed to aid her in the interpretation of the data at no charge. That bioinformatics team then identified a rare heterozygous variant in the *CYP19A1* gene, which encodes aromatase, the rate-limiting enzyme in the synthesis of estrogens from C19 steroid precursors. The variant is a c.358G > C variant in exon 4, encoding the stop codon p.Tyr52X [genomic location chr.15 g.51529196G > C (GRCh37/hg19 Assembly)]. Because this missense variant was predicted to result in a truncation of the enzyme, and because haploinsufficiency for the rate-limiting enzyme in estrogen synthesis might affect circulating estrogen levels or local tissue concentrations of estradiol, clinical correlation was sought in the context of the patient’s medical and family history of polycystic ovarian syndrome.

At that time, UBC’s clinical genetics unit had made an informal policy decision to accept referrals for anyone documented to have carrier status for a rare genetic disorder, whether ascertained DTC or clinically. A local clinical geneticist (W.T.G.) agreed to a session with the consultand to provide genetic counseling for the rare variant found in aromatase, based on the information available at the time. Review of the existing literature identified several individuals reported to be affected with estrogen synthase deficiency, though details on clinically unaffected heterozygous carrier mothers were too sparse to have real clinical utility. At that single clinical visit, we decided that accurate genotype-phenotype correlation would require Sanger sequencing confirmation of the presence of the rare variant in *CYP19A1*, as well as family studies, but that these could not be supported by publicly funded healthcare. We offered to transition the woman’s counseling to a “research-track” (managed by W.T.G. and S. vdB, in consultation with Y.S. and S.J.S. where necessary) and to provide these additional tests under a research protocol at no charge to her. She agreed to this plan in the knowledge that the tests and counseling would be “research-grade” and not “clinical-grade,” with the accompanying caveats (e.g. lack of specific accreditation, unknown error rate, unknown turnaround time, etc.). At the first (clinical) session, she also requested that we provide counseling regarding common *CYP* gene polymorphisms detected by the “detoxigenomic panel” offered by the first DTC company she had contacted. We declined this request because of the unknown analytical validity and accuracy of the test results and confined our counseling to the aromatase variant. Approximately 1 year later, 23andMe provided the consultand with their own analysis of her exome, free of additional charge.

### Clinical Assessment

The consultand’s personal history was as outlined above. On exam, she had no dysmorphic features. Her weight was 80.9 kg and her height was 151.4 cm, for a calculated Body Mass Index of 35.3 kg/m^2^.

The three generation family tree, as reported by the consultand, is presented in Fig. 1. Her parents are thought to be remotely consanguineous; both are of Sinhalese descent from Sri Lanka. Both are on lipid lowering and blood pressure medication. Overall, there is a family history of type 2 diabetes, myocardial infarction, elevated serum cholesterol, skin cancer, hypertension, menorrhagia and polycystic ovarian syndrome. Many family members suffer from chronic pain, anxiety and depression. All of her family members have multiple ongoing health complaints that significantly impact their daily functioning. The consultand has investigated her ancestry on her own and through SNP genotyping provided by 23andMe. She noted that many distant relatives suffered from health problems similar to hers, and often did not have children.

### Sanger Validation of the Consultand’s Rare Variant in Aromatase

Because many rare variants detected by next-generation sequencing methods are false-positives, best practice recommendations are to verify the presence of such variants using an independent method, such as traditional Sanger sequencing (Yang et al. 2013). We designed our own in-house primers to validate the presence of this *CYP19A1* variant in an independently-collected sample from the consultand, and to determine if a genotype-phenotype correlation might be made by tracking the variant through family members affected and unaffected by polycystic ovarian syndrome. All tested family members gave informed consent for these sequencing studies. The variant was confirmed in the consultand, but was not found to co-segregate perfectly with the polycystic ovarian syndrome (PCOS) phenotype within the family (Fig. 1).

### ACMG Recommended Gene Analysis

When clinical-grade WES or whole-genome sequencing is required for patients seen at British Columbia Children’s Hospital or British Columbia Women’s Hospital, our clinical unit currently outsources such testing to accredited clinical labs outside the province, at significant cost to the public health care system. One long-term goal of our center is to offer clinical-grade WES and/or WGS “in-house,” which will require the development of sufficient in-house expertise in interpreting these data. Therefore, in addition to the workup of the *CYP19A1* variant, we attempted to follow the *ACMG Recommendations for reporting of incidental findings in clinical exome and genome sequencing* (Green et al. 2013) by looking for rare variants in the 57 genes believed most likely to harbor actionable mutations. Although these recommendations were not designed to apply specifically to research labs, we decided it was best to attempt to follow these recommendations because we wished to provide interpretation as close to clinical-grade interpretation as was scientifically and economically feasible. The consultand expressed interest in (and gave consent to) being informed of any and all findings in these 57 genes.

When analyzing this individual’s exome data for variants in the genes recommended by ACMG, we found 50 variants in 26 of the 57 genes. These variants were subsequently checked in dbSNP (build 138), 1000genomes, ClinVar and HGMD professional (as of November 25, 2013). Fifteen of the variants were classifiable as being of unknown significance, 26 were predicted to be benign and 7 had mixed reports in terms of their pathogenicity, with insufficient evidence to be clinically actionable. Two variants were predicted to be damaging and potentially clinically relevant. As recommended in the guidelines, these were discussed with the consultand.

The *LDLR* gene, associated with familial hypercholesterolemia (Fokkema et al. 2005), was found to have the p.Glu250Lys variant, which was not found in dbSNP, 1000genomes or ClinVar, but was reported in the LDLR locus-specific database and HGMD database (as of November 25, 2013). Yu et al. (2002) suggested this variant is pathogenic based on its location in a crucial region within the protein, and on its identification among members of a cohort ascertained on the basis of familial hypercholesterolemia. However, no functional experimentation has been published on this specific variant. As we had done for the *CYP19A1* variant, we validated the presence of the *LDLR* variant using in-house primers and Sanger sequencing. We identified the variant in the proband and in several of her family members, some of whom had not yet had lipid levels measured. Even though we could not definitively conclude that the familial *LDLR* variant was causative of the proband’s hyperlipidemia, we offered counseling to family members and recommended that the consultand and all of her first-degree relatives consult with their family physician(s) about having their fasting lipids tested. We made this recommendation to all first-degree relatives, whether or not we had identified the variant in their DNA, because such screening was justified on the basis of the clinical and family history alone. We considered this *LDLR* variant to be a VUS according to the ACMG criteria, though if the category “possibly pathogenic” (stronger evidence for pathogenicity than a VUS but weaker than “probably pathogenic”) were available we would consider it as such.

The *APOB* gene was identified to have a potentially pathogenic variant in our own analysis of the data, and was also noted in the report provided to the consultand by 23andMe. This variant (rs1042031) encodes a missense mutation, p. Glu4181Lys. It is listed as having unknown significance in dbSNP and was predicted to be benign by the 1000genomes database. It was predicted by PolyPhen and SIFT to be benign. HGMD associated the variant with ischemic stroke and aortic stenosis in adults, and with increased APOB levels in the Indian population. Other rare variants in the gene are thought to cause familial hypercholesterolemia, although rs1042031 itself has not been proven to be a major Mendelian mutation causative of familial hypercholesterolemia. We did not pursue Sanger validation of this variant, because the incremental benefit of DNA testing was believed to be minimal in light of the ready availability of serum lipid levels as a screen for hypercholesterolemia and hyperlipidemia, and the fact that we had already counseled the consultand regarding familial hyperlipidemia. Like the *LDLR* variant, we considered this *APOB* variant to be a VUS according to the ACMG criteria, recognizing that it would be almost impossible to separate out the allele-specific effect of the *APOB* variant from that of the *LDLR* variant without a population-level sampling of other individuals of similar ancestry and genotype on whom lipid levels were available.

We also identified a rare variant in the *VWF* gene (rs188526581), encoding a p.Asn1231Ser missense mutation. Although not part of the ACMG recommended panel of genes, we did discuss this variant with the consultand in light of her known clinical diagnosis of von Willebrand disease. We did not pursue Sanger validation of this variant because we believed that there would be no incremental value of such testing for the consultand. She has no plans to reproduce, and a clinically-validated test for circulating vWF levels is already available to her first-degree relatives should they experience signs or symptoms of von Willebrand disease. We considered this *VWF* variant to be “probably pathogenic” according to the ACMG criteria.

### Review of DTC Company Reports

When counseling the consultand, we relied primarily on the analysis performed by the Genome Sciences Centre in Vancouver (Y.S. and S.J.S.). We also examined the unofficial reports, provided to us by the consultand, from two other bioinformatics centers, as well as the “detoxigenomic” SNP result that initiated the consultand’s interest in genetic testing. Lacking detailed knowledge of 23andMe’s variant-calling algorithms and other filtering protocols, we decided that we would counsel based our own bioinformatics analysis of the exome data and selected validation tests (Sanger sequencing and clinical serum tests performed previously). 23andMe provided the consultand with a report listing 37 selected variants, a portion of which is shown in Table II. This company report noted the “predicted effect” of each variant, based solely on the type of mutation (missense, nonsense, silent). The consultand declined publication of the full 23andMe variant report, because of the theoretical risk that such a profile of rare variants might constitute a DNA fingerprint that could be used by third parties to identify her unambiguously.

**Table II:** Selected variants from 23andMe report and Genome Sciences Centre analysis

Gene	Gene Name	Location/Genotype	AA change	Mutation Type, zygosity	dbSNP	1K Genomes	Genotype quality 23andMe/BCGSC	Total Coverage depth at Site 23andMe/BCGSC	Coverage of Variant Allele at Site 23andMe/BCGSC	Predicted effect 23andMe/BCGSC	OMIM
PKHD1	Polycystic kidney and hepatic disease 1 (autosomal recessive)	Chr.6: g.5,1915,066C>A	R723L	NSC, het	rs150597050	0.0006	99.00/228	82/79	NR/29	Moderate/VUS†	606702
LDLR	Low density lipoprotein receptor	chr.19: g.11,224,019G>A	E250K^*^	NSC, het	NA	NA	99.00/193	138/132	NR/62	Moderate/VUS†	606945
NF1	Neurofibromin 1	Chr17: g.29,552,252A>G	K328R	NSC, het	NA	NA	99.00/NR	150/NR	NR/NR	Moderate/Probably benign^a^	613113
TRPS1	trichorhinophalangeal syndrome 1	Chr8: g.116,632,108G>A	H60Y	NSC, het	NA	NA	99.00/228	81/79	NR/35	Moderate/Probably benign^a^	604386
VWF	von Willebrand factor	Chr12: g.6,128,892T>C	N1231S	NSC, het	rs188526581	NA	99.00/44	19/19	NR/8	Moderate/Probably Pathogenic†	613160
APOB	Apolipoprotein B	Chr2: g.21,225,753C>T	E4181K	NSC, het	rs1042031	0.153	NR/112	NR/82	NR/40	NA/VUS†	107730
CYP19A1	Cytochrome P450, family 19, subfamily A, polypeptide A	Chr15: g.51,529,196G>C	Y52X	NSC, het	NA	NA	NR/228	NR/97	NR/43	NA/VUS†	107910

*NR* not reported, *NSC* non-synonymous coding, *VUS* variant of unknown significance

^*^Protein nomenclature and amino acid numbering vary depending on which start codon is deemed canonical by the reporting database

†Classification not based on 23andMe’s classification, but rather from information from the Genome Sciences Centre and using ACMG guidelines for classification. Prediction is based on several factors including PolyPhen and SIFT predicted effect obtained from Variant Effect Predictor, Ensembl version 74

^a^not reported by Genome Sciences Centre; however based on the consultand’s lack of clinical features and family history, it is unlikely to be pathogenic

### Summary of Genetic Counseling

With respect to the initial reason for consultation, we classified the *CYP19A1* variant as a VUS for polycystic ovarian syndrome and counseled the consultand that we could not be certain whether this rare variant had any effect on her overall health, on her unusual number of adverse reactions to medications, or on her polycystic ovarian syndrome. Based on existing literature we considered it to be pathogenic for estrogen synthase deficiency, but since the consultand was heterozygous we considered her to be a carrier of an autosomal recessive disorder and not affected. The difference between the reason for consultation (polycystic ovarian syndrome) and the disease associated with the gene by other studies (autosomal recessive estrogen synthase deficiency) illuminates an emerging theme in the classification of rare variants: pathogenicity (or lack thereof) may need to be defined in a disease-specific way for genes that can be associated with multiple phenotypes (e.g. *APOE*, *LMNA*, the androgen receptor, etc.). The consultand was distressed for a brief time regarding the implications of what she had read online regarding the phenotype of children born with estrogen synthase deficiency. We reassured her that she was not at risk for this and that it would be exceptionally rare for other branches of the family to be affected unless they were to marry a blood relative.

With respect to the *VWF* variant, we considered her to be affected with autosomal dominant von Willebrand disease. We counseled her that the genetic testing explained why she had this phenotype, but that it did not offer additional information relative to her health status beyond what had already been found via standard hematological assays. With respect to the *LDLR and APOB* variants, we counseled her that one or the other of these variants might explain her hyperlipidemia, and that family studies were advisable. However, we specifically counseled her that these studies were advisable on the basis of the lipid testing that she had already had and the known risk to first-degree relatives of someone diagnosed with hyperlipidemia. When she asked us to review the rare variants reported by the company that we had not validated ourselves (see Table II), we counseled her that this report was of limited health value, because the company had only provided a list of “selected” variants without describing their selection criteria. We counseled her that, on the basis of her history and the limited physical examination that we had done, many of these rare variants were clinically irrelevant. For example, she had no cutaneous tumors or facial features of trichorhinophalangeal syndrome, so the rare variants reported in *NF1* and *TRPS1* were likely benign variants or possibly false positives. When we pointed out the description “polycystic kidney and hepatic disease 1 (autosomal recessive)” associated with the PKHD1 variant, she said “that’s really scary.” She rapidly began contextualizing the kidney and liver disease within a genetic framework, leaping to the conclusion that a change in this gene could be the cause. We reassured her that even if this variant were present, at worst we would interpret her clinical status as being an unaffected carrier of an autosomal recessive disease. We said that we did not believe her existing liver disease had anything to do with this *PKHD1* variant (and indeed, the hepatic ultrasound ordered for other reasons prior to the DTC report had found fatty infiltration but no cysts).

Clearly, not all of the rare variants flagged by the company report were false positives, because the company report did identify the *VWF* variant and the *LDLR* variant. The *LDLR* variant was numbered using a different cDNA numbering system than that used by the Genome Sciences Centre, which again illustrates a difficulty that may present itself to clinicians who undertake interpretation of DTC reports. The company report did not identify the (now Sanger-validated) *CYP19A1* variant, though it was clearly identifiable in our interpretation of the company data provided to us by the consultand in .bam format.

## Discussion

We were consulted by a woman who had undergone Whole Exome Sequencing (WES) from a direct-to-consumer (DTC) company, and who was willing to share with us the data and reports provided by the company so that we could offer something approximating clinical recommendations based on these results. This situation provided us with a unique opportunity in genetic counseling, as it mimicked a scenario frequently discussed as a hypothetical: an individual who seeks medical and/or genetic advice regarding the actionability of a DTC company’s reports. This situation also provided us with a unique challenge: that of meeting our fiduciary duty to the consultand at a time when such duties have yet to be defined rigorously. Indeed, our perception of our own fiduciary duties shifted during the time of the project itself – from a very limited duty to interpret the medical consequences of the rare variant in *CYP19A1* to a broader duty to search her variant file for rare variants in the ACMG’s list of actionable genes once their position statement was published (Green et al. 2013). In an attempt to avoid incurring additional costs to our province’s publicly-funded health care system, we avoided repeating tests that had already been performed clinically (i.e. vWF levels and lipid levels).

We made it clear from the beginning that our involvement was for the validation of the *CYP19A1* mutation and that we did not have the resources to validate every rare variant identified by our own analysis or that of the DTC company. To Sanger-validate each of these would take many hours (Ormond et al. 2010) and the design of multiple primer sets. Although we did not specifically review each individual variant with the consultand, we explained the evaluative process on which we determine whether a variant is pathogenic from primary literature. After a thorough search of the literature on *CYP19A1*, we ultimately concluded that the best interpretation of her *CYP19A1* result was that she was a carrier of autosomal recessive aromatase deficiency. There are limited data in the literature on the phenotypes of mothers of children affected with autosomal recessive aromatase deficiency, and no specific mention was made regarding whether any had manifested irregular menses, partial estrogen deficiency or polycystic ovarian syndrome. As a result, we were not able to reach a definitive conclusion on the pathogenicity of rare variants in *CYP19A1* for polycystic ovarian syndrome. Though it is intuitively appealing that truncating mutations in an enzyme such as this one would be a rare but major risk factor for a common disease like polycystic ovarian syndrome (Deladoey et al. 1999; Lafranco et al. 2008; Lin et al. 2007; Pepe et al. 2007; Zirilli et al. 2008), it is important that exome sequencing results (especially DTC exome sequencing results) not be over-interpreted on the basis of intuitive appeal.

The variant reported provided to the consultand by 23andMe provided details on 37 variants selected out of the many thousands that were identifiable in the .bam file. The report on rare variants provided by 23andMe to the consultand listed the location and nature of the rare variants, but provided no interpretation regarding their pathogenicity. Instead, it used a very rudimentary classification system that binned variants into those having “mild,” “moderate” and “high” impact. The company’s research report did include the explicit disclaimer that it was not comprehensive and was provided “for information purposes only,” though the language used elsewhere in the results could easily have been confused with a report that was intended to identify disease-causing alleles. For example, a rare variant in *PKHD1* was reported as occurring in the gene for “polycystic kidney and hepatic disease 1 (autosomal recessive).” The Exome 80X Pilot program was a research pilot advertised as “suitable for customers who are comfortable managing and understanding raw genetic data” (“Exome 80x Pilot project”, 27Sept2011), so it is not surprising that the report was not curated to the extent that would be expected of a clinical exome. Nonetheless, a printed report of this nature carries a risk of eliciting anxiety in the mind of a recipient who is not intimately familiar with the analysis of genomic data (as this consultand was not) (Egglestone et al. 2013). For example, the mention of *polycystic kidney and hepatic disease 1* (*autosomal recessive*) caused the consultand to become concerned, considering the context of her family’s history of kidney and liver disease as well as her own (see Table I).

The company report also flagged rare heterozygous variants in *NF1* and *TRPS1*, which would, if truly present and functional, cause autosomal dominant disorders (neurofibromatosis (OMIM #162200) and trichorhinophalangeal syndrome (OMIM # 190350), respectively). However, it was clear from her medical history and physical exam that neither disorder was present.

Review of various online databases (including locus-specific databases) may provide some indication of a variant’s pathogenicity, but it is also important to consider the consultand’s ancestral or ethnic group. A common method of assessing pathogenicity is the presence of the variant of interest in affected consultands and its absence from healthy individuals (MacArthur et al. 2014). However, a rare benign variant may be inaccurately classified as pathogenic if one assumes that its low prevalence is indicative of pathogenicity (Ackerman et al. 2003). As was counseled in the case report by Sturm and Manickam (2012), pathogenicity of a variant cannot be assumed if the ethnic group in which a variant is reported as pathogenic is different from that of the consultand. As no current Sinhalese reference genome exists, we encountered an additional challenge in interpreting our consultand’s variants.

The consultand initially approached us with a strong belief that the cause of her health problems could be found within her genome. She fit some aspects of the description of an “early adopter” (McBride et al. 2009), but not others. She had no university-level science courses in her educational history and did not have a high level of prior genetic knowledge (although she was eager to learn about and to discuss her results). Additionally, while early adopters are typically more interested in their future susceptibility to common disease, our consultand was looking for genetic causes for her current medical problems, and for those affecting her immediate family members. Despite our caution that genetic causes for common, complex disease are unlikely to be found within her genome, the consultand strongly attributed her multiple health issues to a genetic cause. She attributes her history of unusual reactions to medications to genetic variants in *CYP450* enzymes; this attribution had been strengthened by previous DTC testing of polymorphisms in *CYP450* enzymes.

When examining the ACMG’s list of genes, we found two rare variants (in *LDLR* and *APOB*) as well as one in *VWF* to be potentially pathogenic in the heterozygous state. The consultand’s personal history of von Willebrand disease and her personal and family history of hyperlipidemia did assist us in making actionable recommendations, though a specific causal relationship between these variants and the conditions could not be established without functional studies that would have required site-directed mutagenesis, subcloning and cell culture work. Although we did recommend screening for von Willebrand disease and hyperlipidemia among her first-degree relatives, such family screening was already advisable on the basis of her own prior history of low plasma von Willebrand factor and elevated plasma lipids. The fact that our recommendations were made primarily on the basis of existing clinical diagnoses calls into question the cost-effectiveness and incremental benefit of WES in cases similar to hers (Dorschner et al. 2013).

The boundaries of the fiduciary duty for counseling consultands on DTC company genetic testing results (panel or whole exome sequencing) are unclear. Our team had direct access to sophisticated bioinformatics and to Sanger sequencing in a research setting, tools which are not commonly available to family physicians or to genetic counselors in the community. Such sessions need to be educational for the consultand so that they leave the session equipped to do independent additional and future interpretation (Sturm and Manickam 2012). Although requests to interpret a SNP panel are more common than WES (and involve fewer variants than WES), these requests will still take a considerable amount of time and bioinformatic expertise.

## Conclusion and Lessons Learned

From the very beginning, we entered into a therapeutic contract with the consultand where we were clear and specific on what we could provide in terms of interpretation. For example, we declined to interpret pharmacogenetic variants that might influence the metabolism of flutamide, since specific well-accepted data are lacking in the primary literature to justify this sort of genotype-phenotype correlation. We also declined to interpret every variant in the context of every disease present in the family because disentangling the individual contributions of each variant to common, complex diseases such as type 2 diabetes, depression and fatty liver is currently impossible. We focused instead on answering clearly-predefined questions regarding the potential contribution of the *CYP19A1* variant to polycystic ovarian disease (through genotype-phenotype correlations). When the new ACMG guidelines were published, we recontacted the consultand and updated our therapeutic contract and informed consent to include Mendelian variants potentially causative of otherwise undiagnosed adult-onset disease.

Requests by consultands for interpretation of DTC company genetic testing are still infrequent. We caution health care professionals regarding reports provided by labs that are not certified for medical testing, because consultands will likely expect that any relevant information that is not included in the report itself will be supplied by the interpreting counselor or physician (Howard and Borry 2013). Supplying this information is a time-consuming process, requiring an average of 23–54 min per variant for a trained genetics professional (Dewey et al. 2014; Yang et al. 2013), exclusive of any validation studies that may be required.

It must be borne in mind that individuals who request interpretation of a DTC exome or other DTC test are very likely to have a pre-existing belief that significant, actionable answers will be found in their genomic results. If they did not hold such a belief, it is unlikely that they would have paid for the test in the first place. We believe that public dissemination of successes in identification of genes for rare Mendelian diseases has enhanced expectations in the minds of many members of the public that DNA testing can find “the gene for” their disease. Repeated uses of the phrase “the gene for” in multiple media reports may have led them to expect that genetic contributors to common diseases are identifiable using means similar to those used for rare Mendelian diseases. The mismatch between the expectations of a consultand and what actionable results a health-care provider can feasibly extract from a SNP profile or an exome may lead to frequent disappointment in the DTC genetic testing setting. We would advise genetic counselors to be very cautious in accepting requests to examine DTC data, due to the volume of results and the large amount of work that is required to be certain of the accuracy of each result. For example, the sheer number of variants identified in an exome precludes independent Sanger validation of each rare or common variant.

Throughout this article, we have used the term “consultand” rather than “patient,” in order to make a distinction between individuals who seek interpretation for medically-certified tests (i.e. patients) and individuals who seek interpretation for the results of uncertified tests (i.e. consultands). The latter situation is not a medical process (though it looks very much like it) and such consultations should take place outside of publicly-funded health care systems, such as in a research setting or a private-pay scenario. Our experience with this case convinces us that such consultations are not cost-effective within a publicly funded health-care system, particularly if independent clinical Sanger validation would be required for every variant. Worthy of note is the fact that the DTC exome report provided by the company to the proband was explicitly labeled “for information purposes only.” However, we believe that interpretation of rare variants within the exome is intrinsically a medical testing process. The fact that the company report included mention of polycystic kidney and hepatic *disease* (our italics) and of medical terms like neurofibromatosis and trichorhinophalangeal syndrome means it cannot be considered to contain information that is strictly non-medical in nature.

The US Food and Drug Administration (FDA) has recently issued a warning letter to 23andMe regarding their direct-to-consumer genetic testing, ordering the company to cease marketing the aspects of their SNP genotyping that could be confused with medical testing by members of the public (Gutierrez 2013). This move by the FDA is applauded by some (Annas and Elias 2014) and deemed too harsh by others (Green and Farahany 2014). 23andMe has complied with this order (Gutierrez 2014) and had not, to our knowledge, ever marketed DTC exomes outside of their time-limited Exome 80X Pilot Program which was closed to enrollment prior to the FDA warning. Because of the potential for confusion with medical testing, we regard the reporting of rare variants in OMIM genes (i.e. genes confidently known to be associated with Mendelian disease) as being intrinsically a medical process, one that should be conducted only through the auspices of a medically certified lab or a research study approved by an independent research ethics board. Should private companies like 23andMe seek to enter the DTC exome space, we believe it is most advisable that they seek first clinical certification, engage experienced bioinformaticians, molecular geneticists, clinical geneticists and genetic counselors on their staff, and issue reports that incorporate detailed interpretation of the pathogenicity (or lack thereof) of the rare variants they identify.

## Comments

This manuscript has been submitted solely to the Journal of Genetic Counseling and has not been published elsewhere. The manuscript’s contents have not been previously published and are not anticipated to be published elsewhere.
